# Challenges in surgical and perioperative care for Brazil's aging population

**DOI:** 10.1016/j.bjane.2025.844586

**Published:** 2025-01-14

**Authors:** Andre P. Schmidt, Federico Bilotta

**Affiliations:** a Hospital de Clínicas de Porto Alegre (HCPA), Serviço de Anestesia e Medicina Perioperatória, Porto Alegre, RS, Brazil; b Santa Casa de Porto Alegre, Serviço de Anestesia, Porto Alegre, RS, Brazil; c Hospital Nossa Senhora da Conceição, Serviço de Anestesia, Porto Alegre, RS, Brazil; d Universidade Federal do Rio Grande do Sul (UFRGS), Programa de Pós-graduação em Ciências Pneumológicas, Porto Alegre, RS, Brazil; e Universidade Federal do Rio Grande do Sul (UFRGS), Programa de Pós-graduação em Ciências Cirúrgicas, Porto Alegre, RS, Brazil; f Faculdade de Medicina da Universidade de São Paulo (FMUSP), Programa de Pós-Graduação em Anestesiologia, Ciências Cirúrgicas e Medicina Perioperatória, São Paulo, SP, Brazil; g Sapienza University of Rome, Department of Anesthesiology, Critical Care and Pain Medicine, Rome, Italy

Brazil is undergoing a profound demographic transformation. Recent data from the Brazilian Institute of Geography and Statistics (IBGE) reveals that the proportion of people aged 65 and older has risen sharply, now constituting 11% of the population.^1^ This aging demographic brings with it an increase in chronic degenerative diseases, including cardiovascular conditions, cancer, and neurodegenerative disorders. Coupled with advancements in surgical techniques and anesthetic care, more elderly patients are undergoing complex surgical procedures, often with an increased risk of postoperative complications. Addressing these challenges requires a strategic, patient-centered approach to surgical and perioperative care.

The aging process is accompanied by physiological and functional declines that increase vulnerability to perioperative risks. Elderly patients often present with comorbidities, frailty, and reduced functional capacity, factors that significantly influence surgical outcomes.^2^ Accurately identifying high-risk patients is critical for tailoring perioperative strategies and mitigating adverse outcomes. Risk stratification tools, such as the American Society of Anesthesiologists’ Physical Status (ASA-PS) classification and the Surgical Outcome Risk Tool (SORT) model, are commonly used to predict perioperative risks.^3^ However, their applicability to Brazil's unique patient population, healthcare disparities, and resource constraints remains limited. For example, while tools like the Revised Cardiac Risk Index (RCRI) and ACS-NSQIP calculator offer valuable insights, they have been developed in high-income countries and may not capture the specific challenges faced by low- and middle-income countries (LMICs) like Brazil.^4^ Studies, such as the Ex-Care risk model developed in Brazil, show promise in adapting risk prediction to local realities but require further validation and refinement to ensure broad applicability.^2^, ^3^, ^4^, ^5^

The elderly population is at increased risk for postoperative complications, including major adverse cardiac events, acute kidney injury, respiratory failure, and infections.^6^^,^^7^ Emergency surgeries and prolonged operative times exacerbate these risks. Furthermore, frailty and reduced physiological reserves complicate recovery, often leading to extended hospital stays and increased mortality rates.^8^ Notably, patients with advanced age are also at higher risk for cognitive decline, as corroborated by a population-based study showing that cumulative surgeries are associated with cognitive decline and neurodegeneration.^9^ These findings highlight the importance of preoperative risk stratification for cognitive decline risk factors and prioritizing perioperative brain health, particularly for patients undergoing multiple procedures.^10^^,^^11^ The implementation of prehabilitation might be among the initiatives that can take place in adapting perioperative clinical practice to the aging population in Brazil.^11^ This reality underscores the need for enhanced perioperative care pathways tailored to high-risk older patients.

The preoperative evaluation of older surgical patients extends far beyond traditional cardiovascular assessments, encompassing a comprehensive evaluation of cognitive function, mental health, fall risk, functional deficits, and frailty.^12^ Careful medication review to minimize polypharmacy and inappropriate prescriptions is crucial for preventing complications. Older adults also face heightened risks of cardiac, pulmonary, thromboembolic, and renal complications after surgery. For instance, the risk of postoperative pulmonary complications, such as pneumonia and respiratory failure, can be reduced through aspiration precautions, incentive spirometry, and early mobilization.^13^ Preventing venous thromboembolism with pharmacologic prophylaxis and mechanical compression devices is vital, particularly in high-risk scenarios like malignancy or trauma surgery. Attention to renal function, avoiding nephrotoxic drugs, and optimizing volume status are key to reducing acute kidney injury risk. Postoperatively, targeted interventions are essential to address common geriatric complications, such as delirium, falls, malnutrition, pressure ulcers, and urinary tract infections.^13^^,^^14^ These complications not only increase the immediate perioperative risk but also contribute to long-term adverse outcomes, including cognitive decline, functional deterioration, and diminished quality of life. Strategies to prevent hypothermia, falls, and functional decline, such as fall risk assessments, early mobilization, and enhanced rehabilitation programs, play a central role in optimizing postoperative outcomes for older patients.^14^ Therefore, smooth transitions of care after surgery are critical to preventing hospital readmissions and ensuring sustained functional recovery. By adopting a holistic and proactive approach, perioperative teams can significantly improve the safety and quality of surgical care for Brazil's aging population.

Emerging evidence supports the implementation of high-risk surgical bundles, which encompass comprehensive care models designed to optimize outcomes for vulnerable surgical populations.^15^ These bundles include preoperative optimization, advanced intraoperative monitoring, and enhanced postoperative surveillance, emphasizing early detection of complications and timely interventions. Such pathways, which incorporate measures like perioperative troponin monitoring, intensified postoperative vigilance, and structured communication protocols, offer a promising framework to address the unique challenges of Brazil's aging population. By reducing preventable deaths and improving patient experiences, high-risk surgical bundles represent a critical advancement in perioperative care.

However, the allocation of postoperative care resources remains a significant challenge, particularly in low- and middle-income countries like Brazil. A recent study highlights the association between postoperative intensive care allocation and mortality in high-risk surgical patients within a Southern Brazilian hospital.^16^ The research, which utilized the Ex-Care risk model revealed no independent mortality reduction from intensive care unit (ICU) allocation compared to the postanesthetic care unit (PACU) for high-risk patients. Notably, those in the highest Ex-Care risk class exhibited markedly elevated mortality risks, underscoring the need for adaptive postoperative care solutions beyond traditional ICU settings. This evidence points to a critical gap in resource allocation strategies and the importance of refining risk assessment tools to guide decisions effectively. Addressing these unmet needs through innovative care pathways and evidence-based frameworks could significantly improve outcomes for Brazil's high-risk surgical population.

Postoperative complications significantly affect mortality, slow functional recovery, reduce quality of life, and place a heavy burden on healthcare resources. Comprehensive Geriatric Assessment (CGA) offers a systematic approach to mitigate these risks, with evidence showing reductions in postoperative complications and cost-effectiveness in both elective and emergency surgeries.^17^, ^18^, ^19^ Despite these advantages, implementing CGA in Brazil faces hurdles, such as a limited geriatric workforce, the need for interdisciplinary collaboration, and challenges in adapting CGA methodologies to local contexts.

The COVID-19 pandemic has further highlighted the vulnerabilities of Brazil's older surgical population. Prolonged waiting times for elective surgeries have worsened the progression of underlying conditions and functional decline, while disruptions have increased emergency presentations with advanced pathologies.^20^ Addressing these issues requires a patient-centered, whole-pathway approach to perioperative care, including proactive health screenings, optimizing waiting periods for preoperative preparation, and prioritizing surgeries based on clinical needs. Enhancing traditional perioperative pathways to accommodate older patients with multimorbidities and geriatric syndromes is essential. Integrating geriatric expertise, fostering cross-specialty collaboration, and leveraging national audits and shared best practices are crucial for ensuring high-quality, patient-centered surgical care for Brazil's aging population.

Brazil's public health system, *Sistema Único de Saúde* (SUS), is facing mounting pressures to meet the demands of an aging population. Healthcare disparities, limited access to specialized care, and regional inequities in resource allocation pose significant barriers to delivering high-quality surgical and perioperative care.^21^^,^^22^ Addressing these challenges necessitates coordinated efforts from both public and private sectors. Investment in specialized geriatric surgical care is essential. Establishing outpatient clinics focused on the elderly, expanding laboratory and rehabilitation services, and training medical professionals in geriatric care are critical steps. Additionally, promoting healthy aging and preventive measures to mitigate chronic diseases can alleviate some of the burden on tertiary care facilities.

Brazil's aging population demands urgent attention from policymakers, healthcare providers, and researchers. The integration of validated risk stratification tools, coupled with enhanced perioperative care models, holds the potential to transform surgical outcomes for elderly patients. However, achieving this requires addressing healthcare disparities, investing in workforce training, and fostering collaboration across sectors. As Brazil navigates the complexities of this demographic shift, the focus must remain on patient-centered care that prioritizes safety, quality, and equity. The challenge is formidable, but with targeted interventions and a commitment to innovation, Brazil can rise to meet the needs of its aging population while ensuring better surgical and perioperative outcomes for all.

## Conflicts of interest

The authors declare no conflicts of interest.

## References

[1] Instituto Brasileiro de Geografia e Estatística (IBGE). 2022 Census: Number of elderly persons in the Brazilian population grew 57.4% in 12 years. Available at:https://agenciadenoticias.ibge.gov.br/en/agencia-news/2184-news-agency/news/38187-2022-census-number-of-elderly-persons-in-the-brazilian-population-grew-57-4-in-12-years. Accessed on [date].

[2] Gutierrez CS, Passos SC, Castro SMJ (2021). Few and feasible preoperative variables can identify high-risk surgical patients: derivation and validation of the Ex-Care risk model. Br J Anaesth.

[3] Schmidt AP, Stefani LC. (2022). How to identify a high-risk surgical patient?. Braz J Anesthesiol.

[4] Stefani LC, Gutierrez CS, Castro SMJ (2017). Derivation and validation of a preoperative risk model for postoperative mortality (SAMPE model): An approach to care stratification. PLoS One.

[5] Passos SC, de Jezus, Castro SM, Stahlschmidt A (2024). Development and validation of the Ex-Care BR model: a multicentre initiative for identifying Brazilian surgical patients at risk of 30-day in-hospital mortality. Br J Anaesth.

[6] Gong S, Qian D, Riazi S (2023). Association between the FRAIL scale and postoperative complications in older surgical patients: a systematic review and meta-analysis. Anesth Analg.

[7] Becerra-Bolaños Á, Hernández-Aguiar Y, Rodríguez-Pérez A. (2024). Preoperative frailty and postoperative complications after non-cardiac surgery: a systematic review. J Int Med Res.

[8] Lin HS, Watts JN, Peel NM, Hubbard RE. (2016). Frailty and postoperative outcomes in older surgical patients: a systematic review. BMC Geriatr.

[9] Taylor J, Robledo KP, Medel V (2024). Association between surgical admissions, cognition, and neurodegeneration in older people: a population-based study from the UK Biobank. Lancet Healthy Longev.

[10] Mevorach L, Forookhi A, Farcomeni A, Romagnoli S, Bilotta F. (2023). Perioperative risk factors associated with increased incidence of postoperative delirium: systematic review, meta-analysis, and Grading of Recommendations Assessment, Development, and Evaluation system report of clinical literature. Br J Anaesth.

[11] Crişan I, Slankamenac K, Bilotta F. (2024). How much does it cost to be fit for operation? The economics of prehabilitation. Curr Opin Anaesthesiol.

[12] Mohanty S, Rosenthal RA, Russell MM, Neuman MD, Ko CY, Esnaola NF. (2016). Optimal perioperative management of the geriatric patient: A best practices guideline from the American College of Surgeons NSQIP and the American Geriatrics Society. J Am Coll Surg.

[13] Kumar C, Salzman B, Colburn JL. (2018). Preoperative assessment in older adults: a comprehensive approach. Am Fam Physician.

[14] Wolfe JD, Wolfe NK, Rich MW. (2020). Perioperative care of the geriatric patient for noncardiac surgery. Clin Cardiol.

[15] Stahlschmidt A, Passos SC, Cardoso GR (2022). Enhanced peri-operative care to improve outcomes for high-risk surgical patients in Brazil: a single-centre before-and-after cohort study. Anaesthesia.

[16] Stahlschmidt A, Passos SC, Cardoso GR (2024). Postoperative intensive care allocation and mortality in high-risk surgical patients: evidence from a low- and middle-income country cohort. Braz J Anesthesiol.

[17] Partridge JS, Harari D, Martin FC, Dhesi JK. (2014). The impact of pre-operative comprehensive geriatric assessment on postoperative outcomes in older patients undergoing scheduled surgery: a systematic review. Anaesthesia.

[18] Partridge JSL, Moonesinghe SR, Lees N, Dhesi JK. (2022). Perioperative care for older people. Age Ageing.

[19] Chen L, Zong W, Luo M, Yu H. (2024). The impact of comprehensive geriatric assessment on postoperative outcomes in elderly surgery: a systematic review and meta-analysis. PLoS One.

[20] Miziara ID, Carmen Silvia Molleis Galego M. (2021). Patients waiting lists and the COVID-19 pandemic: A moral dilemma. Perioper Care Oper Room Manag.

[21] Bastos LBR, Barbosa MA, Rosso CFW (2020). Practices and challenges on coordinating the Brazilian Unified Health System. Rev Saude Publica.

[22] Paim J, Travassos C, Almeida C, Bahia L, Macinko J. (2011). The Brazilian health system: history, advances, and challenges. Lancet.

